# Avian Influenza A Virus in Wild Birds in Highly Urbanized Areas

**DOI:** 10.1371/journal.pone.0038256

**Published:** 2012-06-27

**Authors:** Josanne H. Verhagen, Vincent J. Munster, Frank Majoor, Pascal Lexmond, Oanh Vuong, Job B. G. Stumpel, Guus F. Rimmelzwaan, Albert D. M. E. Osterhaus, Martin Schutten, Roy Slaterus, Ron A. M. Fouchier

**Affiliations:** 1 National Influenza Center and Department of Virology, Erasmus Medical Center, Rotterdam, The Netherlands; 2 Laboratory of Virology, Division of Intramural Research, National Institute of Allergy and Infectious Diseases, National Institutes of Health, Hamilton, Montana, United States of America; 3 Sovon Dutch Centre for Field Ornithology, Nijmegen, The Netherlands; University of Georgia, United States of America

## Abstract

Avian influenza virus (AIV) surveillance studies in wild birds are usually conducted in rural areas and nature reserves. Less is known of avian influenza virus prevalence in wild birds located in densely populated urban areas, while these birds are more likely to be in close contact with humans. Influenza virus prevalence was investigated in 6059 wild birds sampled in cities in the Netherlands between 2006 and 2009, and compared with parallel AIV surveillance data from low urbanized areas in the Netherlands. Viral prevalence varied with the level of urbanization, with highest prevalence in low urbanized areas. Within cities virus was detected in 0.5% of birds, while seroprevalence exceeded 50%. Ring recoveries of urban wild birds sampled for virus detection demonstrated that most birds were sighted within the same city, while few were sighted in other cities or migrated up to 2659 km away from the sample location in the Netherlands. Here we show that urban birds were infected with AIVs and that urban birds were not separated completely from populations of long-distance migrants. The latter suggests that wild birds in cities may play a role in the introduction of AIVs into cities. Thus, urban bird populations should not be excluded as a human-animal interface for influenza viruses.

## Introduction

Wild aquatic birds are frequently infected with influenza A viruses. Wild birds are assumed to be the original source of influenza A viruses currently circulating in the animal and human population, as wild birds are often infected with all known influenza A virus hemagglutinin (H1–H16) and neuraminidase (N1–N9) subtypes [1], [2]. In most cases wild birds are infected with low pathogenic avian influenza (LPAI) viruses that cause no or only mild disease symptoms in their natural hosts. LPAI viruses can occasionally be transmitted to domestic bird and mammalian species in which they can cause mild to severe disease. Since the first discovery of influenza A viruses in wild birds in 1961 (A/Tern/South Africa/1961) [3], wild birds have been monitored for the presence of influenza A viruses [4], [5]. However, wild bird sampling activities were intensified [6] after the emergence of highly pathogenic avian influenza (HPAI) H5N1 viruses in South-East Asia, and the detection of HPAI H5N1 viruses in migrating wild birds since 2005 [7]–[9]. The increase of wild bird sampling activities worldwide resulted in the expansion of the number of sampled species and locations, with most species sampled belonging to the orders *Anseriformes* (ducks, geese and swans) and *Charadriiformes* (shorebirds and gulls). In addition to the early detection of HPAI viruses, these studies are important to understand the global circulation of both HPAI and LPAI viruses [10]. In most cases, avian influenza virus (AIV) surveillance studies in wild birds were conducted in rural areas and nature reserves characterized by low human densities. AIVs, including HPAI viruses, have sporadically been reported from wild birds in highly urbanized areas [11]–[13], but very little is known about the frequency of AIV infection in wild birds in cities and the risk these birds could pose to domestic animal and human health. Since 2007 the majority of the global human population is more urban than rural, and the number of people living in urbanized areas is expected to continue growing in the next decade [14]. In many countries, highly urbanized areas contain canals and large city parks with ponds, housing a wide variety of wild and semi-domesticated wild birds. We hypothesized that AIVs are present in wild aquatic birds present in these cities, with prevalence varying with the level of urbanization. We further hypothesized that wild birds sampled near closed water bodies (stagnant water, not connected to other water sources) will be infected with AIV, suggesting these birds play a role in the introduction of AIVs into cities. Here we addressed the questions whether wild aquatic birds present in cities are infected with AIVs and if so, if viral prevalence corresponds with the level of urbanization and connections with closed and open waters.

**Table 1:** Avian influenza prevalence and seroprevalence in wild bird species sampled in highly and low urbanized areas in the Netherlands between 2006 and 2009.

	Highly urbanized areas^1^				Low urbanized areas^2^			
Species	Virology		Serology		Virology		Serology	
	Sampled	Virus positive (%)	Sampled	Seropositive (%)	Sampled	Virus positive (%)	Sampled	Seropositive (%)
Mallard	515	10 (1.9)	101	66 (65.3)	14080	1181 (8.4)	34	21 (61.8)
Egyptian Goose	122	0	7	3 (42.9)	298	4 (1.3)	0	0
Black-headed Gull	3789	16 (0.4)	98	34 (34.7)	3653	270 (7.4)	78	38 (48.7)
Common Gull	609	2 (0.3)	81	68 (84.0)	65	0	6	6 (100)
Lesser Black-backed Gull	479	1 (0.2)	1	0	72	0	1	0
Herring Gull	314	1 (0.3)	17	9 (52.9)	325	8 (2.5)	3	2 (66.7)
Common Coot	231	0	43	3 (7.0)	167	0	10	1 (10.0)
**Total**	**6059**	**30 (0.5)**	**348**	**183 (52.6)**	**18660**	**1463 (7.8)**	**132**	**68 (51.5)**

1 >1500 addresses/km^2^.

2 <1500 addresses/km^2^.

## Methods

Cloacal and oropharyngeal swabs and blood samples were collected from free-living birds in highly urbanized areas - defined here as cities with >1500 addresses per km^2^ -, in the Netherlands from 2006 to 2009. In most cases birds were located in city parks in close proximity to surface waters, in mixed age and species groups. Most sample locations were described either as being located in the centre or in the periphery of a highly urbanized area, and/or being located near open flowing water (in connection with larger water facilities, e.g. canals) or closed stagnant water (not connected to other water sources, e.g. city park ponds). Ducks, geese, gulls and coots were captured by an experienced ornithologist, either individually using a rope with a loop, or with multiple birds at one time using a clap net. All sampled birds were marked individually with a metal leg ring, and bird movements were recorded based on the recoveries of these bands. For comparison of the data obtained from the highly urbanized areas, we used data collected during ongoing AIV surveillance studies in rural, low urbanized areas with little human activity in the Netherlands during the same years. An independent Animal Ethics Committee of the Erasmus Medical Center (Stichting DEC Consult) approved these studies (permit number 122-09-20), in accordance with national and international guidelines. RNA was isolated from cloacal and oropharyngeal samples and analyzed using a real-time reverse transcriptase-PCR (RRT-PCR) assay targeting the matrix gene. All matrix RRT-PCR positive samples were used for detection of H5 and H7 influenza A viruses by using hemagglutinin (HA) specific RRT-PCR tests and for virus isolation in embryonated chicken eggs as described elsewhere [15], [16]. The HA subtype of virus isolates was characterized using a hemagglutination inhibition assay and the neuraminidase (NA) subtype was determined by RT-PCR as described [15]. Blood collected from the brachial vein of birds was centrifuged at 3000 g for 10 minutes in 0.8 ml gel separation tubes (MiniCollect® tubes, Roche). Serum was tested in a multispecies blockingELISA specific for the nucleoprotein (NP) of influenza A viruses (IDEXX FlockChek* AI MultiS-Screen) according to the manufacturers instructions.

To test the statistical significance of the results the Chi-square test, or the Fisher’s exact test if appropriate, was performed using the software from the R project for statistical computing.

## Results

### Avian Influenza Virus and Antibody Detection in Wild Birds in Cities

Cloacal and oropharyngeal samples were collected from 6059 wild birds of 7 species in highly urbanized areas. During the same years, samples were collected from 18660 birds of the same 7 species in rural areas (Table 1). Birds were sampled year round in both highly and low urbanized areas, but in highly urbanized areas the largest proportion (65%) of samples was obtained in January, November and December, while in low urbanized areas the largest proportion (49%) of samples was obtained in June, September and October. The number of sampled hatch year (HY) and after hatch year (AHY) birds were distributed equally in high and low urbanized areas, with the exception of HY Black-headed Gulls that were intensively sampled in June and July at their breeding colonies in rural areas. In highly urbanized areas, influenza A viruses were most frequently detected by RRT-PCR in Mallards (*Anas platyrhynchos*). Less frequently, viruses were detected in Black-headed Gulls (*Chroicocephalus ridibundus*), Common Gulls (*Larus canus*), Herring Gulls (*Larus argentatus*), and Lesser Black-backed Gulls (*Larus fuscus*), and no viruses were detected in Egyptian Geese (*Alopochen aegyptiaca*) and Common Coots (*Fulica atra*) (Table 1) in highly urbanized areas. No viruses of the H5 subtype were detected, and one LPAI virus of the H7 subtype was isolated. Viruses were isolated from 5/30 RRT-PCR positive samples, including viral subtypes H6N8, H7N1, H11N1 and H11N9. In rural areas, influenza A viruses were most frequently detected in Mallards and Black-headed Gulls. Less often, viruses were detected in Common Gulls, Herring Gulls, and Egyptian Geese, and no viruses were detected in Lesser Black-backed Gulls and Common Coots in low urbanized areas. Major differences in virus prevalence between birds in highly and low urbanized areas were found in Mallards, Black-headed Gulls and Herring Gulls only (P<0.05).

**Figure 1:**
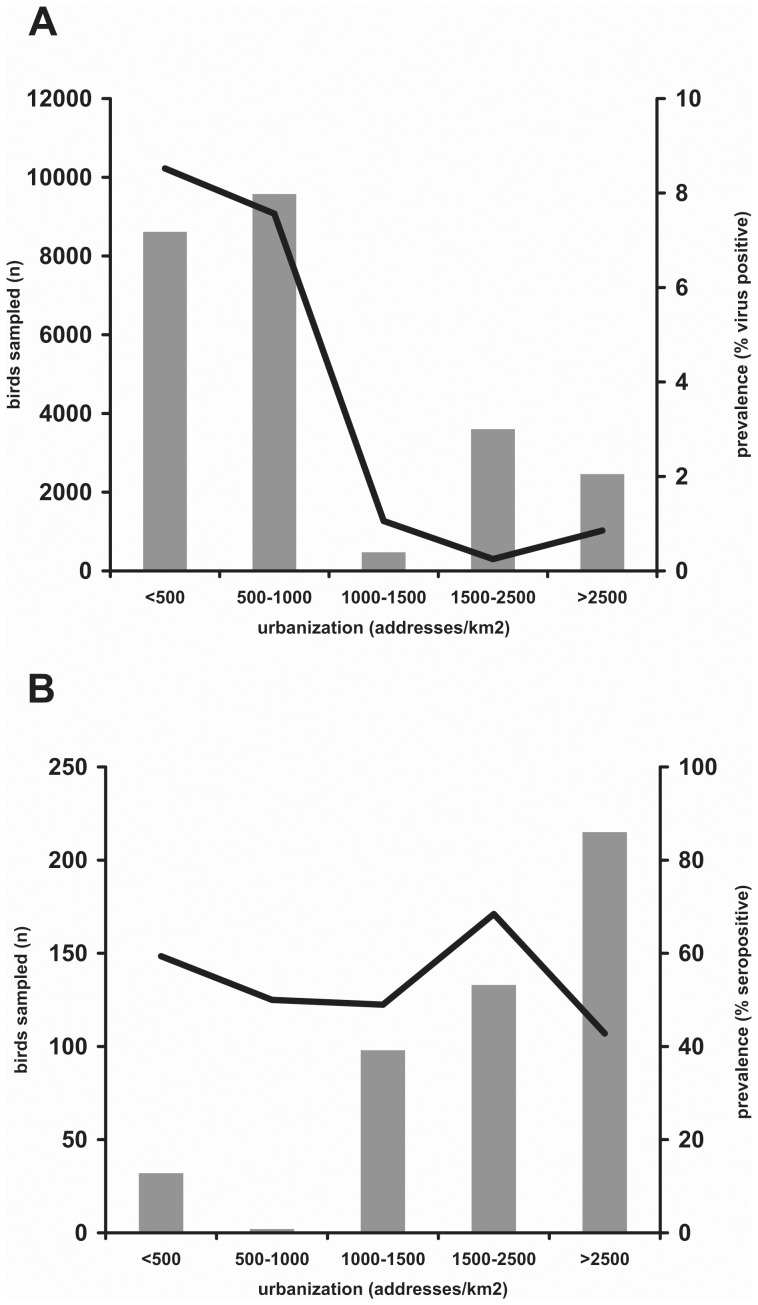
Prevalence of avian influenza virus and antibodies in wild birds based on level of urbanization. Avian influenza virus prevalence (A) and seroprevalence (B) in 7 wild bird species sampled in the Netherlands between 2006 and 2009 in relation to the level of urbanization. Grey bars indicate number of birds sampled (left Y-axes) and triangles indicate prevalence (right Y-axes).

Overall, influenza A virus antibodies were detected in 183/348 (52.6%) of birds sampled in highly urbanized areas, and in 68/132 (51.5%) of birds in rural areas (Table 1). In highly and low urbanized areas, antibodies were detected in 8/50 (16.0%) and 5/15 (33.3%) of HY birds respectively, while antibodies were detected in 175/298 (58.7%) and 63/117 (53.8%) of AHY birds (P>0.05). Thus the seroprevalence in highly and low urbanized areas was similar. In contrast to the seroprevalence data, virus detection rates decreased with increasing levels of urbanization (Figure 1). Nevertheless, avian influenza viruses were even detected in the centers of densely populated cities, in 29/3264 (0.9%)of birds tested.

### Role of Migrating Urban Birds in Introduction of Avian Influenza Viruses in Cities

430 birds of 6 different species sampled in cities were subsequently sighted on various locations. Of the 430 sighted birds 300 birds (69.8%) were only reported back at the same location as where they were ringed initially and 94 birds (21.9%) were sighted at different water bodies in the same city. However, 5/206 Mallards, 6/45 Egyptian Geese, 2/123 Common Coots, 10/37 Herring Gulls, 11/11 Common Gulls and 2/8 Lesser-Black backed Gulls (36/430 birds (8.4%)) migrated between cities and remote areas. The most extreme cases were Common Gulls and Mallards ringed in cities in the Netherlands that were reported back up to 1125 km away in Lithuania and 2659 km away in Russia, respectively. These data indicate that the populations of long distance migrants and birds in urbanized areas are connected and that migrating populations may introduce avian influenza viruses into densely populated urban areas. In agreement with this suggestion we found that influenza viruses were even detected in 21/1847 (1.1%) birds living in closed water bodies in cities thus excluding the possibility of introduction of influenza virus by water flow.

## Discussion

In highly urbanized areas in the Netherlands, AIVs were found to circulate in ducks and gulls. Although the overall AIV prevalence in highly urbanized areas was significantly lower as compared to rural areas, it was certainly not negligible. In addition, most Mallards in rural areas were sampled in September and October during virus peak prevalence in this species, while most Mallards in highly urbanized areas were sampled in November when virus prevalence was decreasing. If more Mallards in cities were sampled more intensively during virus peak prevalence, possibly more viruses would have been detected in urban Mallards. We show that the AIV prevalence was inversely correlated with the level of urbanization, while AIV seroprevalence was approximately constant for the different levels of urbanization. The latter may suggest that birds in rural and urban areas have similar likelihood of experiencing influenza virus infection at least once, but that birds in rural areas may be exposed more frequently. Although some birds breed in highly urbanized areas, large flocks of immunologically naïve birds most likely primarily aggregate in rural areas whereby facilitating transmission as compared with urban populations that consist more often of single individuals or small groups of a single family.

For AHY Barnacle Geese and White-fronted Geese it was shown that seroprevalence increases with age (unpublished data).Although the level of antibodies in AHY birds sampled in highly and low urbanized areas was similar, it is possible that the group of urban AHY birds consisted of older birds compared with birds sampled in low urbanized areas. Older birds had a longer window of exposure to viruses that may result in a detectable antibody response. It is further possible that birds in urban environments live longer than birds in rural areas because of e.g. high food availability. The availability of food in highly urbanized areas possibly also makes the bird less susceptible to infections, and might leave more energy to produce a strong long lasting antibody response.

Since AIV were detected in birds residing in both closed and open water bodies, we suggest that wild birds rather than water flow acted as vector for introduction of AIV into cities. Indeed, analysis of the movements of the sampled birds indicated that city populations were not separated completely from populations of long-distance migrants, and that populations moved between different water bodies within cities. Together, our data indicate that viral epizootics in wild migrating birds may directly impact bird populations in urbanized areas, and that urban bird populations should not be excluded as a source of influenza virus infection for humans and animals.
